# Supplementary material for: Are carbon emissions trading and green financial instruments synergistic? -Comprehensive quantitative research based on content analysis
Source: PLoS One. 2024 Mar 7;19(3):e0298601. doi: 10.1371/journal.pone.0298601 (PMC10919601; doi:10.1371/journal.pone.0298601)
Supplement: S1 Data — (ZIP) [file pone.0298601.s001.zip › Data/地级市Z_F工作报告.zip!/╔╜╢1⁄2/╫═▓⌐╩╨/└┤╫╘╣1⁄2╓┌║┼í╛┬φ┐╦╩2╛▌═°í┐_2009.docx]

各位代表：

　　现在，我代表市人民政府，向大会作政府工作报告，请予审议，并请各位政协委员和其他列席会议的同志提出意见。一、2008年工作回顾刚刚过去的一年是极不寻常、极不平凡的一年。在中共淄博市委的坚强领导下，各级政府和全市人民坚持以邓小平理论和“三个代表”重要思想为指导，深入贯彻落实科学发展观，积极抢抓机遇，沉着应对挑战，解放思想，同心同德，顽强拼搏，实现了经济平稳较快发展和社会和谐稳定，在殷实和谐经济文化强市建设的征程上迈出了坚实步伐，年初确定的各项任务目标能够全面实现。

　　（一）积极应对宏观形势变化，国民经济保持健康发展。预计全市生产总值完成2300亿元，增长13％以上；固定资产投资增长20％以上；境内财政收入达到24786亿元，其中地方财政收入11469亿元，增长172％；城市居民人均可支配收入和农民人均纯收入分别增长11%和13%以上；金融机构各项存贷款余额分别比年初增加285亿元和137亿元。我们坚决贯彻中央、省一系列重大决策部署，增强经济工作的预见性、针对性，抢抓机遇，积极作为。去年前三季度，面对通胀压力加大、大宗商品价格大幅上扬、煤电油运气供应趋紧的复杂形势，我们正确把握国家“两防”和“一保一控”等宏观调控政策及其调整变化，着力加强生产要素供应保障，及时开辟电煤、成品油供应渠道，用改革的思路化解局部金融风险，加大节能减排工作力度，取得了显著成效。进入第四季度以后，针对日益加深的国际金融危机影响，我们明确把保增长作为经济工作的首要任务，加强分析研判，强化经济运行监测，密切政银企合作，抓关键、保重点，保持了经济平稳运行。预计全年地方工业销售收入、利税、利润分别增长27％、255％和24％。中央出台扩大内需促进经济增长的政策措施后，我们及时组织上报重点项目，其中有总投资近百亿元的建设项目得到中央、省核准备案，部分项目争取到中央和省预算资金支持，做到了出手快、出拳重、措施准、工作实。

　　(二）坚持科学发展不动摇，坚定推进发展方式转变。不论宏观形势如何变化，我们始终坚持科学发展不动摇，积极推进结构调整，大力拓展发展空间。中心城区东部化工区产业布局调整正式启动，南部建材区综合整治取得显著成效。深入开展治污减排、环境保护、土地执法系列专项行动，对一大批存在违法排放和安全隐患的建陶、化工企业以及私开滥挖的小煤井、小矿山企业进行了综合治理。我市的水泥和碳酸钙产能正在实现脱胎换骨式的改造升级。化工异味、含酚废水、粉尘烟尘污染等突出环境问题得到了有效治理。COD、SO2净削减率分别达到509％和85％，全市万元GDP综合能耗、规模以上工业万元增加值能耗能够完成年度目标。着力提高自主创新能力，新增省级以上工程技术研究中心和企业技术中心1７家，总数达到１３８家，中国名牌产品和中国驰名商标分别达到30个和27个。预计高新技术产业产值增长27％，占全市规模以上工业总产值的比重达到336％，比上年提高147个百分点。我市跻身中国城市综合创新力50强和最具创新绩效城市行列。大力发展服务业，启动实施服务业十大重点推进工程，金融保险、文化旅游、现代物流、科技信息等现代服务业实现新发展。预计全市服务业增加值占GDP的比重达到31%以上；社会消费品零售总额完成729亿元，增长23％。

　　（三）坚持城乡统筹，新农村建设扎实推进。坚持“一个主体、两轮驱动”，深入实施“建设农村殷实小康十大工程”，落实支农惠农各项政策补贴209亿元，粮食实现面积、单产、总产三增长，连续六年丰产丰收，马桥小城镇、皇城现代农业等“八大示范片区”初显规模。农业标准化生产基地突破100万亩，规模以上龙头企业达到210家，无公害、绿色、有机食品总数达到141个。农村基础设施建设进一步加强，新建农村公路465公里，新建、改造村村通自来水工程310处，解决和改善了378个村、18万人的饮水安全问题。大力发展农村社会事业，新建规范化卫生室400个，组织实施了农村文化信息共享工程、中小学教学仪器更新工程、现代远程教育工程以及“家电下乡惠农”工程，农村物质文化生活更加丰富。

　　（四）着力打造生态和谐宜居城市，城市功能实现新提升。围绕建立城乡一体的规划体系，启动了全市村镇规划、综合交通规划等20余项重大规划的修编，城市总体规划按法定程序转报国务院审批。突出抓好城建重点项目，市体育中心建设进展顺利，新客运中心开工建设，旧居住区改造全面启动，湖南路、高淄路等干线公路升级改造，昌国路、杏园东路、张南路等城市路网建设项目顺利完成。大力强化生态环境建设，启动了新一轮碧水蓝天行动计划，新改扩建污水处理厂8座，新建雨污管道1047公里，全市污水处理能力达77万吨/日，城市污水集中处理率达到85%，6座污水处理厂出水水质达到一级A排放标准，市垃圾焚烧发电厂基本满负荷运行，城市垃圾无害化处理率达到985％。以前所未有的力度推进秸秆禁烧和综合利用，实现了全面禁烧的目标！启动和实施四宝山、萌山水库生态修复和污染治理、淄河、猪龙河综合整治等一批重大生态建设项目，取得了显著成效。全市新增造林面积116万亩，完成路域水系绿化210公里，新增园林绿地314公顷。社区建设扎实推进。以迎奥运和创建全国文明城市为契机，全面启动新一轮城乡环境综合整治，建立了全覆盖、全时段的中心城区环卫保洁体制，城市形象明显提升。

　　（五）深化改革开放，发展活力日益增强。统筹区域协调发展，调整下放三个老工业区经济建设管理权限，突破高青、支持沂源工作取得新成效。万杰高科实现战略重组，联合化工成功上市，旺达股份实现挂牌交易，鲁泰纺织、鲁阳股份获准增发，7家拟上市公司引进战略投资者。小额贷款公司、村镇银行试点扎实推进，浦发银行淄博支行正式成立，淄博邮政储蓄银行完成组建。财税、投融资、价格等领域的改革稳步推进。努力提高对外开放水平，预计全年实际到位外来投资170亿元左右，比上年略有增长；其中，实际利用外资475亿美元，同比增长105%。实现进出口总额565亿美元，其中出口36亿美元，同比增长20%。陶博会、新材料技术论坛等重大展会活动成功举办。对口支援工作取得新进展。

　　（六）坚持以人为本，民生和社会建设不断加强。始终将促进就业放在保民生的重要位置来抓，就业形势保持了基本稳定。城镇居民医疗保险基本实现全覆盖，新型农村社会养老保险试点顺利启动，国有破产企业退休职工基本医疗保险问题得到全面解决，“五项保险”在全国同类城市中率先实现市级统筹。提高了企业退休人员养老金、在职职工最低工资标准，城乡低保实现应保尽保。累计为7700户城市低收入住房困难家庭实施廉租住房保障，住房公积金对城镇职工购买住房的支持能力进一步增强。全面实施免费义务教育，义务教育巩固率达到100％，高中段教育普及率达到95％；加强教育基础设施建设，新建一批中小学校，城乡办学条件不断改善；高等教育、职业教育、特殊教育水平进一步提高。完善公共卫生和医疗服务体系，新农合参合率达9903%，社区卫生服务体系更加完善。妥善处置“问题奶粉”事件，食品药品监管进一步加强。全民健身工程扎实推进，竞技体育水平不断提高，“两运”筹备工作进展顺利。加强人口计生工作，稳定了低生育水平。成功创建全国红十字服务示范市。国防动员、国防教育、人民防空建设得到加强，双拥优抚安置工作取得新成绩。全面推进文明淄博建设“十大行动”，我市再次创建为“省级文明城市”，被推荐为“创建全国文明城市工作先进城市”。启动文化建设“五大工程”，国家、省“精品工程”获奖数量连续三年居全省首位。把维护社会稳定摆在更加突出的位置，完善社会矛盾预防和化解机制，深化社会治安综合治理，全力做好奥运安保工作，平安淄博建设扎实有效。防灾减灾体系进一步完善，应急管理体制建设扎实推进。毫不放松地抓好安全生产，保持了安全生产形势的稳定。统计、民族宗教、外事、侨务、对台、新闻出版、广播电视、气象、防震、史志、档案、仲裁、老龄、慈善、残疾人等各项事业都实现了新发展。

　　2008年是本届政府任期的第一年。一年来，各级政府按照为民、务实、清廉的要求，扎实开展解放思想大讨论和深入学习实践科学发展观活动，自身建设不断加强。加快转变政府职能，深化行政管理体制改革，更加注重运用经济、法律手段调控经济运行，注重通过创新体制机制改进社会管理和公共服务，行政行为进一步规范，行政效能进一步提高。切实提高决策的科学化、民主化水平，落实目标责任，强化工作督查，加大问责力度，干事创业、狠抓落实的氛围更加浓厚。深入开展“法律五进”活动，大力加强政府法制建设。强化政府监察、审计工作，全面落实廉政建设责任制，政府廉政建设取得新成效。

　　各位代表！过去的一年，我们在保持经济社会平稳较快发展的同时，有效处置重大突发事件，全力以赴支援抗震救灾，淄博的城市精神得到充分彰显，城市形象得到有力提升。年初，积极支援南方抗击雨雪冰冻灾害。面对突如其来的“4·28”胶济铁路特别重大交通事故，迅速启动应急机制，不讲条件、不计代价，全力做好事故救援、伤员救治和善后处理工作，实现了“最大限度减少死亡和因伤致残、妥善处理善后、维护大局稳定”的任务目标，得到了中央领导和省委、省政府的充分肯定，受到社会各界的广泛赞誉。“5·12”汶川特大地震发生后，社会各界迅速行动，举全市之力支援抗震救灾和灾后重建。广大共产党员积极交纳特殊党费，全市各界踊跃捐款捐物，卫生、公安特警、消防、民兵预备役等多支队伍参与前线救援救助。对口援建在全省创出“十一个率先”，活动板房援建效率被总结推广为“淄博速度”。在重大考验面前，全市人民表现出高度的政治觉悟、大局意识和无私奉献精神，这是新时期淄博城市精神的生动写照，也必将成为加快淄博现代化建设的强大动力。

　　各位代表！过去的一年，我们战胜诸多困难和挑战，取得了和谐发展、科学发展的新成就。这是中共淄博市委坚强领导的结果，是全市上下奋力拼搏的结果，是市人大、市政协和社会各界有效监督、大力支持的结果。在此，我代表市人民政府，向为淄博现代化建设做出贡献的全市人民，向各位人大代表、政协委员，向各民主党派、工商联、各人民团体和各界人士，向驻淄部队、武警官兵和中央、省驻淄单位，向关心支持淄博发展的海内外各界朋友，表示衷心的感谢和崇高的敬意!

　　在看到成绩的同时，我们也清醒地认识到,我市经济社会发展和政府工作还存在一些困难和问题。主要是:受国际金融危机影响，部分行业、企业生产经营困难，部分企业被迫关闭或停产半停产，工业经济增幅下滑过快，就业形势十分严峻；农产品价格回落，城市吸纳农村人口就业能力减弱，农民持续增收难度加大；经济结构性矛盾依然突出，发展方式仍较粗放，节能减排压力仍然很大，发展空间亟待拓展；影响社会和谐稳定的因素增多，一些群众关注的热点难点问题尚未得到有效解决；面对困难和挑战，部分政府工作人员的思想观念、服务意识、业务素质、工作作风等不能很好地适应形势发展变化的需要。对这些问题，我们一定高度重视，采取有力措施，切实加以解决。二、2009年面临的形势和主要任务2009年是建国60周年，做好今年工作意义十分重大。

　　今年我市经济社会发展面临的形势相当严峻。国际金融危机仍在蔓延，对实体经济的影响仍在加深，其严重后果还会进一步显现。由于国际经济形势的急剧变化，国内经济运行困难加剧，全球经济衰退与国内周期性调整叠加在一起，使宏观经济形势异常复杂。但也要充分认识到，我国经济发展的基本态势没有改变，我们遇到的困难和挑战是前进中的问题。改革开放３０年，我们形成了比较坚实的物质基础和产业支撑，综合实力、抵御风险能力显著增强，经济结构调整效应开始释放；我们正处于工业化、城镇化快速推进的关键时期，发展潜力巨大，特别是国家和省扩大内需的政策措施对经济发展的拉动更加直接、更加强劲，我们完全有条件、有能力抵御风险，战胜困难，夺取胜利。为此，必须坚定做好今年工作的信心和决心，充分看到危机中蕴含的转机，挑战中存在的机遇，审时度势，迎难而上，科学务实，积极作为，以为化危，以为求机，努力把金融危机的压力，顺势转化为调整结构、自主创新、节能减排、转变发展方式的动力，转化为盘活存量、优化增量、加强项目建设的动力，转化为树立新的资源观、拉长产业链、打造特色产业集群的动力，努力在经济形势的重大变化中抢占发展先机，增创淄博经济社会科学发展的新优势。

　　今年政府工作总的要求是：全面贯彻党的十七大和十七届三中全会精神，以邓小平理论和“三个代表”重要思想为指导，深入贯彻落实科学发展观，着力保增长扩内需调结构，确保经济平稳较快增长，确保统筹城乡实现新突破，确保民生持续改善，确保社会和谐稳定，抓住新机遇，增创新优势，实现新发展，不断开创殷实和谐经济文化强市建设新局面，以优异成绩迎接建国六十周年。

　　全市经济社会发展的主要建议目标是：地区生产总值增长11%左右，地方财政收入增长10%左右，规模以上固定资产投资增长20%左右，社会消费品零售总额增长16%左右，实际利用外资增长10%左右,进出口总额增长10%左右,城市居民人均可支配收入和农民人均纯收入均增长8%左右。主要约束性建议目标为：万元GDP综合能耗降低58%以上，化学需氧量和二氧化硫排放量分别削减45%和8%以上。人口自然增长率控制在55‰以内。

　　实现经济社会发展的目标任务，最根本的是要深入贯彻落实科学发展观，把落实扩大内需方针与淄博实际紧密结合起来，把保增长、上水平、增活力、强后劲、重民生贯穿经济社会发展的全过程，努力实现科学发展、和谐发展、可持续发展。

　　——必须把保持经济平稳较快发展作为首要任务。越是在经济发展面临困难的时候，越是要坚持发展是硬道理的战略思想，牢牢扭住经济建设这个中心不放松。要始终坚持发展第一要务，坚持好字优先、好中求快、又好又快，抓住扩大内需重大机遇，加强重点项目建设，突破经济社会发展的薄弱环节，保证经济平稳较快发展，同时为长远发展打好基础。

　　——必须把扩大内需作为保增长的主要着力点。在国际市场需求明显减少的情况下，必须采取强有力的措施，坚决贯彻国家扩大内需的战略方针。要强化投资拉动，千方百计增加投资和优化投资结构，着力扩大消费需求特别是居民消费需求，充分发挥和强化淄博的比较优势，更好实现内需为主和积极利用外需的共同拉动。

　　——必须把加快发展方式转变和结构调整作为保增长的主攻方向。转变发展方式是实现科学发展的战略选择。在经济调整过程中，必须更加坚定地把结构调整作为主线，把节能降耗作为关键，把环境保护作为“命门”，大力推进自主创新、节能减排和集约节约用地，努力拓展发展空间，坚决防止低水平重复建设，不断增强可持续发展的能力。

　　——必须把深化改革开放作为保增长的强大动力。在经济增速下滑、各方面困难增多的情况下，更要解放思想，深化改革开放，消除体制机制障碍，破解发展难题，激发发展活力。要统筹兼顾，加大重点领域和关键环节的改革力度，妥善处理好改革发展稳定的关系。积极应对激烈的国际竞争，抓住国际分工调整时机，拓展对外开放广度和深度，提高对外开放水平。

　　——必须把改善民生作为保增长的出发点和落脚点。要坚持把维护好、实现好、发展好人民群众的根本利益放在首位，切实落实保障和改善民生的各项政策，真正把扩大投资的民生工程办成群众得实惠的民心工程，着力解决群众最关心、最直接、最现实的利益问题，在经济增速放缓的情况下确保各项民生继续改善，促进社会和谐稳定。三、千方百计扩大内需扩大内需是促进经济平稳较快增长的基本立足点和长期战略方针。必须深入贯彻落实国家和省一系列政策措施，切实强化投资、消费双拉动。一是强化投资拉动。加大项目实施力度，对已经确定实施的项目，抓紧落实土地、资金、能评、环评及基础配套，确保早日开工，尽快形成实物工作量，力求最快的拉动效果。围绕社会民生、基础设施、生态环保、自主创新、现代产业体系等方面，集中实施一批大项目、关键项目。着力抓好高新区六大特色产业园、白杨河等三个大电厂、城乡电网建设改造、地方铁路以及南水北调、西气东输地方配套等一批投资规模大、拉动作用明显的重大项目。按照“实施一批、储备一批、策划一批”的思路，立足当前，着眼长远，通盘考虑今后三年的项目建设，加快项目策划、储备跟进速度，及时调整充实上报项目资源库，有针对性地加大对上争取力度，努力保持项目建设的持续拉动。二是努力扩大消费。千方百计增加居民收入，特别是提高农民和城市中低收入者的收入水平，建立与经济发展相适应的收入增长机制，提高居民消费能力。切实提高社会保障水平，消除制约消费的制度和政策障碍，改善居民消费预期。着力培育新的消费热点，尤其是激发能较快拉动我市产业发展的农民住房建设积极性，力求取得新农村建设、城乡统筹以及增加建设用地空间、拉动相关产业发展多赢的效果。加快推进实施“万村千乡市场工程”和社区商业网点建设工程，新建或改造标准化连锁农家店500个。扩大“家电下乡”政策补贴范围，促进农村家电消费。严厉打击制售假冒伪劣产品和市场欺诈行为，努力改善消费环境。三是促进房地产市场平稳健康发展。全面落实国家、省出台的财税、金融政策，坚持“保住一头、启动一头、放开一头”，促进房地产市场平稳健康发展。“保住一头”就是加快保障性住房建设。在三年内，全面完成400多万平方米的棚户区、老旧工矿区、城中村改造建设任务，其中保障性住房要加快建设实施。“启动一头”就是启动农村房地产发展。结合新农村建设和农村城镇化，鼓励农民建房，拉动固定资产投资。“放开一头”就是对中高档商品房要按照市场化的路子，引导房地产企业创新融资方式，增强发展能力。全面落实国家鼓励居民购房的各项信贷优惠政策，推动城镇居民扩大住房消费。进一步规范房地产市场秩序，促进房地产业健康有序发展。四是建立强有力的项目推进机制。建立健全扩大内需项目推进组织领导机制，围绕项目实施需要，开展工作、创造条件、优化环境。对国家和省明令取消的行政事业性收费我市要坚定不移的贯彻执行。要创造更加优良的金融生态环境，打造好政府与企业的融资平台，全面落实四大国有银行对我市的三年新增贷款600亿元的金融支持计划，争取更多的股份制银行、有实力的境内外证券公司、投资公司、基金公司来我市设立分支机构、开展业务。努力做好政府专项债券发行和企业债券融资工作，积极争取国债资金。积极推动企业上市和上市企业再融资。大力发展风险投资，推进私募股权融资，打造战略投资者投资高地。系统研究、认真贯彻落实国家支持扩大内需项目建设的政策措施，拓宽发展空间，合法高效服务，保障建设项目用地需求。四、促进结构优化升级充分运用客观形成的倒逼机制，加快构筑现代产业体系，促进产业结构优化升级。一是全力培植壮大骨干产业。抓住国家和省实施产业振兴计划的重大机遇，在重大产品开发、重大技术创新、重大项目布局方面加大衔接和争取力度，顺势推进骨干产业做大做强。用好增值税转型改革、进口设备免税等优惠政策，加大石油化工、轻工、建材、陶瓷、冶金、机械等传统产业技术改造力度，发挥重点技术改造导向计划引领作用，力争技术改造投入占到全市固定资产投入的40％以上。加快培育新材料、精细化工、信息、医药、汽车和机电装备等新兴产业。与“双百工程”相结合，加快推进“新、特、优”工程，从支持优势产品入手，推动一批创新成长型企业加快发展。二是切实增强自主创新能力。加快技术创新体系建设，整合科技资源，提升产学研合作水平，推动建立多方共赢的公共创新平台和行业科技联盟。对关键和共性技术，要强化政府扶持力度，连续投入，持续推进，确保实现重大突破。大力发展高新技术产业，探索充分发挥高新区辐射带动功能的有效途径，灵活高效运用高新区平台，使其真正成为全市高新技术产业发展的重要“引擎”，力争高新技术产业产值有较大幅度增长。三是推进企业战略性重组。以国家产业政策为指导，抓住当前国家支持兼并重组的有利时机，搞好重点行业、重点企业的兼并重组。鼓励行业龙头企业、优势企业兼并重组落后企业、困难企业。鼓励优势企业强强联合，对关联企业、上下游企业实行战略重组，提高生产集中度。鼓励有实力的企业开展跨国并购，加快国际化经营步伐。努力争取更多的中央、省属大企业来我市实施资产重组，支持驻淄大企业抓住机遇加快发展。四是加快产业布局优化调整。正确处理我市组群式城市特点与历史形成的产业集群之间的关系，积极稳妥地推进城市建设、产业布局、生态建设“三和谐”。切实抓好东部化工区搬迁改造、张钢环境综合整治等重大产业布局调整项目。强化省级以上经济园区的载体功能，切实搞好土地资源的优化配置和集约利用，加强园区内行业、企业间配套协作，有效拉长产业链，促进主导产业和优势产业集聚发展。五是切实搞好经济运行。强化市场开拓指导，引导企业细分市场，调整产品结构，巩固扩大市场占有率。大力开拓重点项目建设市场，开展直供配送和融资租赁，提高重点工程市内产品配套能力。密切关注金融危机对实体经济的影响，对重点行业以及对就业、利税、财政等有重要影响的重点企业实施动态监控，建立快速反应机制，发现苗头性、倾向性问题，及时采取有效措施积极应对。推动全市企业全面加强内部管理，加快企业制度创新和管理创新。强化对煤电油运的综合平衡和协调调度，搞好生产要素保障。

把服务业发展摆到更加突出的位置。深入实施服务业十大重点推进工程，加大“三大载体”培植力度。高水平策划实施一批服务业发展带动性项目，加快发展现代物流业，打造鲁中“旱码头”；大力发展金融、中介服务、要素市场，建设适应我市产业升级、社会发展的服务业支撑体系。坚持政策引导、招商引资、项目提升相结合，加快文化旅游、科技信息、社区服务等现代服务业发展。推进流通现代化，运用先进信息技术、管理手段、运营模式改造提升传统商贸流通业。五、加快农村改革与发展认真贯彻党的十七届三中全会精神，把加快形成城乡经济社会发展一体化新格局作为根本要求，坚持城乡统筹、以工促农、以城带乡，坚持“一个主体、两轮驱动”，推进城乡生态安全、规划建设、产业布局、基础设施配套、公共服务和社会管理、劳动就业与社会保障等“六个一体化”，不断推动农村经济社会又好又快发展。一是加快发展现代农业。落实最严格的耕地保护制度，确保基本农田保护面积长期稳定。全面完成100万亩高产优质专用粮基地建设。围绕牧菜果三大主导产业，加快基地化建设、标准化生产、产业化经营，大力发展有机农业，培育壮大重点农业龙头企业，提高市场竞争力，增强对种植业、养殖业的带动能力。继续推进烟叶生产生态村建设。加快农技推广、动植物疫病防控、农产品监测及农业综合服务站等农村公共服务体系建设，促进农业发展方式转变。二是千方百计增加农民收入。采取农业内部挖潜、增加农民工资性收入、落实惠农强农优惠政策、加大扶贫开发等多种措施，千方百计增加农民收入。实施“阳光工程”培训计划。扩大政策性农业保险试点范围。全面落实各类涉农补贴政策。扩大扶贫范围，增加扶贫项目，力争使9万农村贫困人口尽快脱贫致富。三是加强农村基础设施建设。加强村镇布局优化调整，加快中心镇、中心村建设，发挥小城镇的吸纳和带动功能。全面完成“百座小型水库”除险加固工程。对大型灌区、中低产田实施集中改造。推进农村饮水安全工程，解决15万人的饮水安全问题。完成新农村电气化改造年度任务。提高农村新能源利用水平，加大秸秆气化、农村沼气池工程建设力度。启动实施村村通油路改造提升工程，改造建设农村公路800公里。实施农村“三清、四改、五化”工程，改善村容村貌。四是深化农村各项改革。稳定农村现行经营体制，允许农民按照依法自愿有偿的原则，发展多种形式的土地规模经营。保持现有土地承包关系稳定并长久不变，积极稳妥地开展土地承包经营权流转试点，探索建立土地流转市场。积极推进集体林权制度改革，加快建立现代林业产权制度。积极开展“一事一议筹资筹劳”试点，建立村级公益事业建设机制。继续推进农村综合改革。六、努力拓展发展空间统筹经济社会发展与资源节约、环境保护，不断拓展发展空间，增强可持续发展能力。一是大力推进节能降耗。切实加强源头控制，搞好能耗评估和审查，对区域能耗水平实行总量控制，对新上高耗能项目实行等量淘汰。要分类制定重点产品能耗标准，有序淘汰落后产能，大力发展循环经济，加强资源综合利用，确保固体废物综合利用率达到90%以上。制定严密可行的减排规划和实施计划，细化责任，狠抓落实，确保“十一五”节能降耗目标任务完成。二是加强环境保护和生态建设。加快实施新一轮碧水蓝天行动计划，推进生态市建设。把深度治理大气污染作为重中之重，突出抓好二氧化硫、化工异味综合治理，全面实施6个“30”工程，完成所有电厂及建陶、钢铁、焦化等大气污染主要行业的二氧化硫治理，对偷排偷放行为实施“顶格处罚”，确保环境空气质量达到省下达的指标要求。切实抓好污水处理设施和配套管网建设，对城市污水处理厂进行全面升级改造，确保城市污水收集率达到95%以上，全市所有污水处理厂出水水质全部达到一级A排放标准。加快中水回用工程建设，完成华能白杨河电厂新机组等中水回用示范工程建设。实施范阳河疏浚治理、萌山水库生态建设、孝妇河改造提升、清水润城、四宝山引水等五大生态水利工程，推进重点流域综合治理和水源地保护，加快实现全市主要水体变清目标。加快环保产业发展，推广清洁生产。巩固提高秸秆禁烧和综合利用成果，切实抓好农村环境保护工作，实现城乡环保工作一体化。三是努力提高土地集约节约利用水平。强化政策落实，切实提高投资强度和建筑容积率，鼓励引导企业利用提高技术水平、加盖多层厂房等方式，在不增加占地的前提下，提高产出效率。制定专门方案，采取集中处置、推动企业间兼并重组等方式，有效盘活存量土地。认真研究国家和省村庄建设用地存量置换政策，积极稳妥地推进旧村改造、合村并居、退二进三、土地功能转换，加大土地整理复垦力度，最大限度地弥补建设用地年度计划指标不足。严格土地执法，及时处理各类土地违法案件，确保土地市场秩序实现根本好转。七、继续深化改革开放改革开放是解决前进中问题的根本出路。必须通过努力，消除机制体制障碍，破解发展难题，扩大对外开放，激发发展活力。

　　加大重点领域和关键环节改革。一是积极推进财税体制改革。调整完善财政管理体制。全面落实增值税转型、燃油税费改革的各项配套政策。积极推进财税资源优化重组，全面推进企业实施二、三产业分离，增加地方财政收入。强化国有资产和政府资源监管运营，完善国有资源有偿使用机制，对土地、矿产、旅游景点、城市公共场地等资源运营，全面实行招标拍卖，不断拓宽财政增收渠道。二是加快推进金融体制改革。积极推进农村中小金融机构改革，加快组建村镇银行，深化农村信用社改革，稳妥有序地推进小额贷款公司试点。市及区县要扩大贷款担保机构规模，增强担保能力，财政收入10亿元以上的区县要有3-5家担保机构。高度重视防范金融风险，完善应对金融风险的体制机制。三是稳妥推进资源性产品价格改革。提高粮食最低收购价。推进清洁能源和可再生资源能源价格改革。积极推进水价综合改革，合理确定各类用水的水资源费标准，制定农业灌溉用水价格改革政策，加大超标准用水定额加价制度推行力度。加快建立健全矿产资源有偿使用制度和生态环境补偿机制。四是继续深化企业改革。鼓励、支持、引导非公有制经济加快发展。积极参与省非上市公司股权托管机构筹建，争取设立分支机构，加强与“三板”市场和天津场外交易市场的对接，为非上市公司股权流通创造条件。加快推进城市供气、供热、公交、污水和垃圾处理等领域市场化改革。五是加快推进行政管理体制改革。深化市与区县管理体制改革，加大对高青、沂源支持力度，支持老工业区科学发展、加速振兴，增强区县域经济发展活力。六是做好省综合配套改革试点工作。争取在相关领域率先闯出路子。

　　坚持走以质取胜、集约化、多元化的路子，努力提高对外开放的质量和水平。一是坚持更高水平的“引进来”。充分发挥我市产业链、产业集群发展潜力大的优势，加强产业招商，打造对外招商新亮点，吸引更多的国内外战略投资者来淄发展。要更加重视引进人才、引进先进技术，推进企业境外上市和承接服务外包。二是加快外贸发展方式转变。坚持让利不让市场的战略思维，引导企业加快调整出口产品结构，在国际市场调整重组、国际资源重新布局中抢占先机。用足用好出口退税、外贸发展基金、财政贴息、国家政策性贷款等政策措施，加大对骨干出口企业的扶持力度，支持拥有自主品牌、核心技术产品和农、轻、纺等有竞争力的劳动密集型产品出口。保住传统市场，加大对新兴市场的开拓力度，促进外贸市场多元化，降低出口风险。引导企业扩大急需的先进技术、设备、关键零部件和能源原材料进口。三是坚持更大步伐的“走出去”。抓住海外投资新机遇，加大我市急需的稀缺资源和关键技术领域的对外合作力度。鼓励支持企业收购矿产资源开发权，开发和加工资源性产品。积极融入全省“一体两翼”发展布局，加强同半岛城市群、省会城市群经济圈的合作与对接，在更高平台和层次上聚集发展要素，释放发展潜能，拓展发展空间。扎实做好支援北川县灾后重建工作。八、加快生态和谐宜居城市建设坚持“城乡统筹，一体化发展”，切实把握“四个原则”、“八个意识”，抓住国家扩大内需机遇，加快推进城市化进程。一是坚持规划先行。加快推进城乡规划无缝隙覆盖。强化城市规划与新一轮土地利用总体规划的有机衔接，围绕统筹配置城乡建设用地，加快把中心城区、次中心城区周边新农村住房建设纳入城区建设规划，把县城周边新农村住房建设纳入县城建设规划，把中心镇周边新农村住房建设纳入中心镇建设规划。在规划中通盘考虑居住、生活、卫生、教育、产业、基础设施等合理布局，强化社区服务功能，整体推进城乡建设。二是加快推进城市产业布局调整。加快中心城区东部化工区搬迁改造进度；提前完成四宝山地区生态修复任务，提高绿化标准和基础设施建设水平，打造城市近郊体育休闲公园；提升南部建材区和市高新区、桓台开发区产业布局规划发展水平，与城市建设有机融合。三是加强城市基础设施建设。抓住国家扩大内需、实施积极的财政政策和适度宽松的货币政策这一重大机遇，充分发挥政府主导作用，在城市交通、体育文化、旧城区改造、教育卫生等方面，启动实施一批城建重点项目，力求这批工程既能迅速形成实物工作量，又能形成城市新的亮点工程。突出抓好治污减排债券项目，提高城市生态建设水平。提前完成我市生态市建设计划任务，在应对国际金融危机过程中有效拉动我市相关产业发展。四是提高城市管理水平。巩固提高社区在城市管理中的基础地位。逐步完善全市数字化城市管理信息系统，提高城市管理的信息化、精细化水平。继续实施城乡环境综合整治，改善城乡环境。九、推动文化大发展大繁荣大力发展文化事业和文化产业，推动文化创新，加快由文化资源大市向文化强市跨越。一是加强社会主义核心价值体系建设。把社会主义核心价值体系融入国民教育和精神文明建设的全过程，筑牢全市人民共同奋斗的思想基础。以创建全国文明城市为目标，大力弘扬“诚信、务实、开放、创新”的新时期淄博城市精神，深入开展群众性精神文明创建活动，坚定理想信念，加强舆论引导，创作优秀作品，为经济社会发展提供思想保证、舆论支持、文化支撑。二是加快构建覆盖城乡、惠及全民的公共文化服务体系。加大公益性文化事业投入，全面实施乡镇（街道）综合文化站、社区文化中心、农村文化大院、乡村少年宫建设和升级改造工程。着手规划建设市图书馆新馆等一批重点文化设施，增强公共文化服务功能。深化文化体制、公益性文化事业单位改革，激发文化事业发展活力。三是大力发展文化产业。整合地域文化资源，强化载体建设，提高品牌影响力和带动力。加快建设齐文化产业园，做好“田齐王陵”、“齐国故城遗址”、“桐林遗址”整体申遗工作。打造“淄博陶瓷，当代国窑”品牌。加快培育文化产品市场和要素市场，切实抓好会展、文博、现代传媒、工艺美术、动漫创意、影视演艺、文化用品等文化产业，不断扩大文化产业规模。四是抓好“两运”协办和承办工作。切实搞好场馆建设、服务保障、安全保障、备战参赛等各方面工作，组织开展“全民健身与全运同行”主题活动，以良好的精神风貌当好东道主。十、着力改善民生把保障和改善民生放在更加突出的位置，扎实办好学习实践活动确定的１８件实事，着力增强改善民生的普惠性，切实解决影响民生的突出问题。一是实施更加积极的就业政策。今年就业形势十分严峻，必须实施更加积极的就业政策，强化政府投资和重大项目带动就业的作用，多渠道开发就业岗位，千方百计扩大就业容量。加强大学生就业指导和服务，鼓励大学生自主创业。加强职业技能培训，加大对下岗人员和农民工的免费培训力度，鼓励和支持困难企业加强职工转岗或在岗培训，加强对企业裁员行为的监控力度，防止出现大规模集中裁员。充分发挥创业带动就业的倍增效应，全面落实税收优惠、小额担保贷款、资金补贴、场地安排等创业扶持政策，激发全社会创业活力。深入开展就业援助工作，将就业援助对象扩大到“4050”人员及其他下岗失业困难人员，帮助就业困难群众、特困家庭大中专毕业生以及零就业家庭实现稳定就业，确保实现零就业家庭当年“动态清零”。二是完善社会保障制度。巩固新型农村基本养老保险试点成果，按照“先保后征”原则，优先吸纳被征地农民参加基本养老保险。提高新农合和城镇居民基本医疗保险财政补助标准。将在校大学生纳入城镇居民基本医疗保险范围。对“五大保险”实施扩面征缴，把更多非公有制企业、个体工商户和灵活就业人员纳入社会保障范围。提高企业退休人员养老金水平，建立市级机关事业单位退休人员津贴补贴统筹制度。对城市居民最低生活保障实行分类施保，农村低保标准由900元提高到1000元，并做到应保尽保。积极支持红十字、老龄、慈善、残疾人等事业发展。三是加快教育事业发展。坚持教育优先，加快城区中小学布局调整，新建扩建一批城区中小学校，尽快解决班额过大、孩子就近上学难的问题。完善义务教育经费投入保障机制，启动中小学办学条件标准化建设，加强农村中小学校舍改造，实施“两热一暖一改”工程。完善贫困家庭大中专及普通高中学生资助政策体系。着力提高职业教育发展水平。大力实施人才战略。四是提高医疗卫生保障水平。深化医疗卫生体制改革，建设覆盖城乡居民的医疗服务体系和公共卫生体系。新农合政府补助标准提高到80元。全面完成农村卫生室和城市社区卫生服务机构建设任务，加快市中心医院、第一医院病房楼改扩建等重点卫生基础设施建设，着力解决和改善群众看病贵和就医难的问题。继续做好人口计划生育工作，不断完善利益导向政策体系，稳定低生育水平。五是切实加强社会管理，努力维护社会稳定。继续扎实推进“平安淄博”建设，落实“稳定第一责任”，全面实施“固本强基维稳”工程，及时排查化解苗头性、倾向性问题，牢牢把握维稳促和工作主动权。综合运用法律、经济、行政等手段，努力把矛盾纠纷化解在基层、消除在萌芽状态。大力加强社会治安综合治理，实施治安安民工程，依法严厉打击各种违法犯罪活动。认真抓好安全生产，强化行政问责，有效遏制重特大安全生产事故发生。加大食品药品生产、流通监管力度，严厉打击制售假劣食品药品行为。加快推进应急平台体系建设，提高预防和处置突发公共事件的能力。加强价格监测，维护市场稳定，保障居民生活必需品的市场供给。深入开展国防教育，加强民兵预备役和人防工程建设，大力推进双拥共建。搞好和谐社区建设，共建共享美好家园。进一步做好统计、民族宗教、外事、对台、侨务、仲裁、新闻出版、史志、档案、气象、防震减灾等工作。

　　各位代表！面对国际金融危机，实现今年经济社会平稳健康发展，任务艰巨，责任重大。各级政府必须更加注重自身建设，以改革创新的精神和认真专业务实的作风，研究新情况、把握新规律、解决新问题。一是要切实提高组织领导科学发展的能力和水平。始终坚持以解放思想为先导，努力把深入学习实践科学发展观活动取得的成果转化为谋划科学发展的思路、领导科学发展的实际能力、促进科学发展的具体措施。要科学判断形势，增强战略思维，提高工作预见性和前瞻性，牢牢把握工作主动权。要抢抓机遇，顺势而为，善于在形势变化中把握机遇，在政策调整中抓住机遇，集中精力抓落实，专心致志保增长。二是坚定不移地推进科学行政、民主行政、依法行政。牢固树立正确政绩观，改革完善政府决策机制，规范决策程序，强化决策责任，提高决策水平，努力创造经得起群众、实践和历史检验的政绩。强化政务公开，切实保障人民群众的知情权、参与权、表达权、监督权，提高政府工作透明度和公信力。深入学习贯彻《国务院关于市县政府依法行政的决定》，牢固树立依法行政理念，提高依法行政能力。深化行政执法体制改革，减少执法层次，规范行政行为，强化责任追究。认真接受人大及其常委会的法律和工作监督，自觉接受人民政协的民主监督，高度重视司法监督和行政监察、审计监督，积极推动新闻舆论监督和社会公众监督，努力建设“阳光政府”。三是切实转变工作作风。把群众需求作为第一信号，把维护群众利益作为第一追求，把群众满意作为第一标准，多做群众急需的事，多做群众受益的事，多做打基础的事，多做增后劲的事。要躬身实践，问计于民，深入基层，深入群众，真诚倾听群众呼声，切实把执政为民的要求融入到思想感情里，体现在政策措施中，落实到具体工作项目上。四是切实加强廉政建设。牢固树立过紧日子的思想，艰苦奋斗、勤俭节约，努力降低行政成本，建设节约型政府。深入开展反腐败斗争，严格落实廉政建设责任制，坚决纠正行业不正之风，严肃查处各类违法违纪案件，依法严惩腐败分子。加强对政府公务员的培训、管理和监督，努力建设一支勤政廉洁、务实高效的公务员队伍，建设让人民满意的政府。

　　各位代表！过去的一年，我们经受住了严峻考验；展望未来，我们充满应对危机、战胜困难的信心。让我们更加紧密地团结在以胡锦涛同志为总书记的党中央周围，坚持以邓小平理论和“三个代表”重要思想为指导，深入贯彻落实科学发展观，在中共淄博市委的坚强领导下，紧紧依靠全市人民，抓住机遇，迎难而上，开拓进取，埋头实干，为加快建设殷实和谐经济文化强市而努力奋斗！
